# A Spatiotemporal Database to Track Human Scrub Typhus Using the VectorMap Application

**DOI:** 10.1371/journal.pntd.0004161

**Published:** 2015-12-17

**Authors:** Daryl J. Kelly, Desmond H. Foley, Allen L. Richards

**Affiliations:** 1 Viral and Rickettsial Diseases Department, Naval Medical Research Center, Silver Spring, Maryland, United States of America; 2 Department of Evolution, Ecology and Organismal Biology, The Ohio State University, Columbus, Ohio, United States of America; 3 Division of Entomology, Walter Reed Army Institute of Research, Silver Spring, Maryland, United States of America; 4 Preventive Medicine and Biometrics Department, Uniformed Services University of the Health Sciences, Bethesda, Maryland, United States of America; University of Queensland, AUSTRALIA

## Abstract

Scrub typhus is a potentially fatal mite-borne febrile illness, primarily of the Asia-Pacific Rim. With an endemic area greater than 13 million km^2^ and millions of people at risk, scrub typhus remains an underreported, often misdiagnosed febrile illness. A comprehensive, updatable map of the true distribution of cases has been lacking, and therefore the true risk of disease within the very large endemic area remains unknown. The purpose of this study was to establish a database and map to track human scrub typhus. An online search using PubMed and the United States Armed Forces Pest Management Board Literature Retrieval System was performed to identify articles describing human scrub typhus cases both within and outside the traditionally accepted endemic regions. Using World Health Organization guidelines, stringent criteria were used to establish diagnoses for inclusion in the database. The preliminary screening of 181 scrub typhus publications yielded 145 publications that met the case criterion, 267 case records, and 13 serosurvey records that could be georeferenced, describing 13,739 probable or confirmed human cases in 28 countries. A map service has been established within VectorMap (www.vectormap.org) to explore the role that relative location of vectors, hosts, and the pathogen play in the transmission of mite-borne scrub typhus. The online display of scrub typhus cases in VectorMap illustrates their presence and provides an up-to-date geographic distribution of proven scrub typhus cases.

## Introduction

VectorMap is a spatial database of vector and host collection records, distribution models, and vector-borne disease (VBD) maps that provide a “one-stop shop” for exploring vector hazard and VBD risks on a global scale. Based on MosquitoMap [1] and building on SandflyMap [2], VectorMap uses ESRI’s ArcMap Server 10 (Redlands, CA) and MS Silverlight (Redmond, WA) to serve distribution data on mosquitoes, sand flies, ticks, fleas, mites, and hosts and reservoirs. As part of the establishment of the new mite vector disease service (aka MiteMap) on the VectorMap Silverlight viewer that maps the location of mite (chigger) collection data, we have endeavored to include published data on scrub typhus distribution. Scrub typhus (aka mite-borne typhus or tsutsugamushi disease) is an acute febrile disease endemic within the countries of the Asia-Pacific Rim. It is caused by the bite of *Orientia tsutsugamushi*-infected *Leptotrombidium* spp. larvae (i.e., chiggers). Scrub typhus is regarded as “probably the single most prevalent, under-recognized, neglected, and severe but easily treatable disease in the world” [3]. In recent years there have been reports of confirmed or probable scrub typhus well outside the classic diagnostic triangle for this disease [4–9]. Based on the results of a literature review, we make available in MiteMap the locations of cases of human scrub typhus after applying stringent diagnostic criteria to categorize probable or confirmed scrub typhus cases from the World War II era to the present. Data from VectorMap will assist investigators in selecting potential sites for vaccine trials, as well as identifying at-risk areas for spraying to control vectors. In addition, the use of VectorMap will assist clinicians who must diagnose and treat cases as endemic areas expand. We aim to: (1) describe and develop a comprehensive updatable case mapping system for gathering human scrub typhus information, (2) propose standards for what constitutes a human case and recommend how it should be reported in the literature, (3) discuss the problem of geolocating transmission sites from case study data, (4) improve our understanding of the global geographic area of transmission (i.e., where is the “endemic” area, and is this a useful concept?), (5) demonstrate the value in mapping cases, and (6) recommend possible directions for future studies of the geography of mite-borne scrub typhus.

## Materials and Methods

### Literature review and case and/or publication selection criteria

An online search using PubMed (NCBI, NIM, NIH, Bethesda, MD) [10], and the United States Armed Forces Pest Management Board Literature Retrieval System (AFPMB) [11] was performed to identify articles describing scrub typhus cases both within and outside the traditionally accepted endemic regions. The search terms were used separately, without Boolean “AND, OR” qualifiers, and searched the article title and key words. Search terms were “scrub typhus,” “tsutsugamushi disease,” “*Orientia tsutsugamushi*,” and “*Rickettsia tsutsugamushi*.” For this initial VectorMap, review articles published in English and for years 1940 through 2015 were selected. Articles used included individual case reports, epidemiological reports, clinical trials, febrile patient “fevers of unknown origin” (FUO) studies, and serosurveys consisting of testing of single or paired sera. The minimum requirements for inclusion of an article was that it should contain sufficient data to score probable and/or confirmed cases by applying standardized diagnostic criteria (Table 1) [12] and some geographic information to enable mapping of the likely patient catchment area.

**Table 1:** Criteria used to score patient diagnosis of scrub typhus applied to each publication.^a^

Probable Case	Clinical Presentation	Eschar at bite site (may be absent), acute fever, headache, conjunctival injection, lymphadenopathy, maculo-papular rash (may not be present or may be missed in patients with dark or sunburned skin), cough (common) defervescence within 24–48 hours of antibiotic (tetracycline, etc.) therapy.
	Laboratory Criteria	(Serology) Detection of specific IgM (EIA at 1:100, IP at 1:32, IFA at 1:10) in a single acute serum sample.
Confirmed Case		A probable case with laboratory confirmation
	Laboratory Criteria	Isolation by inoculation of patient blood into mice or cell culture, or PCR positive, or seroconversion (4-fold rise in serological titer) in paired (acute and convalescent) sera by ELISA, IFA, IP, complement fixation or Weil-Felix OX-K.

^a^Adapted from World Health Organization Recommended Surveillance Standards (WHO/CDS/CSR/ISR/99.2 p.123)

EIA, enzyme immune assay; ELISA, enzyme-linked immunosorbent assay; IFA, indirect fluorescent antibody assay; IP, indirect immunoperoxidase assay; PCR, polymerase chain reaction assay.

### Data screening form

An Excel spreadsheet adapted from earlier entomological descriptions in VectorMap [2] was created. The spreadsheet contained 30 fields, including information about human cases, location data where patients acquired infections or presented to the clinicians, and dates of diagnosis from each “source” or reference. Several references identified multiple case presentation sites and dates. In these instances, each site was scored as a separate record.

### Criteria used to determine diagnoses

The criteria that were used to score data from the literature as probable (suspected) or confirmed were adapted from World Health Organization Recommended Surveillance Standards [12]. Data included published studies involving anonymous reviews of hospital records. A georeference (decimal latitude and longitude) was calculated based on text location descriptions using Geonames (http://www.geonames.org/), Google Maps (https://maps.google.com/), Google Earth (http://www.google.com/earth/), and internet search engines. The resulting georeference can be visualized as the anchor point or centroid of a zone (circle) of uncertainty (error), where the disease transmission site is likely to occur. The point radius method [13] was used to portray error as a radius in meters around a geocoordinate; in the case of a likely patient catchment area for a clinic or hospital, the resulting circle can be very large. Various sources of error contribute to the radius of the error around a georeference [14], and the MaNIS Georeferencing calculator (http://manisnet.org/gci2.html) was used in some instances. Foley et al. [15] discussed many of the methodological aspects associated with georeferencing in an arthropod vector context.

## Results

This literature review was not intended to be exhaustive, but rather was meant to identify and present a subset or cross section of reports from the majority of geographic areas and years, including confirmed and probable human scrub typhus cases.

**Fig 1:**
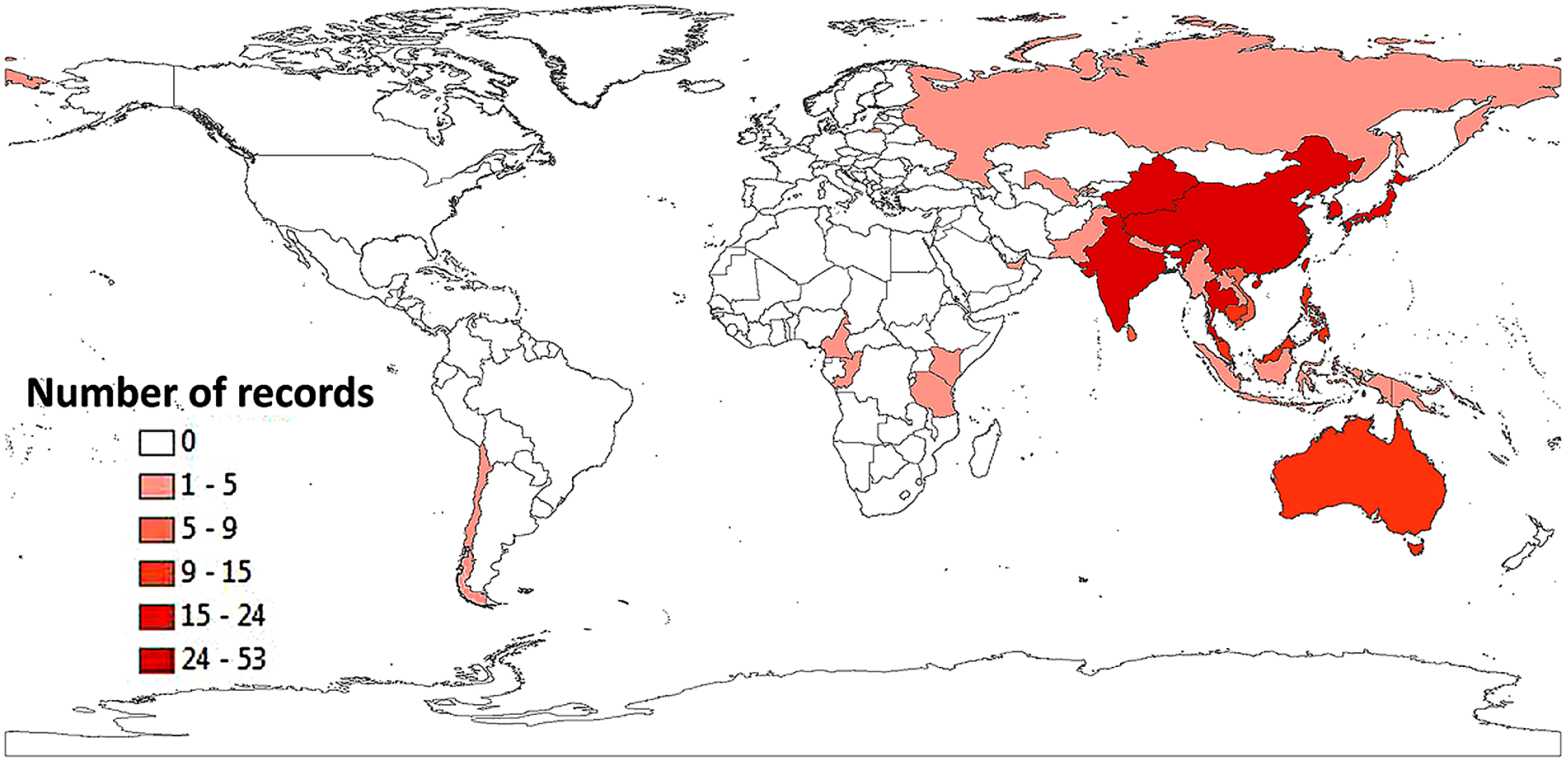
Map showing numbers of records by country of scrub typhus cases from the literature included in this study.

**Fig 2:**
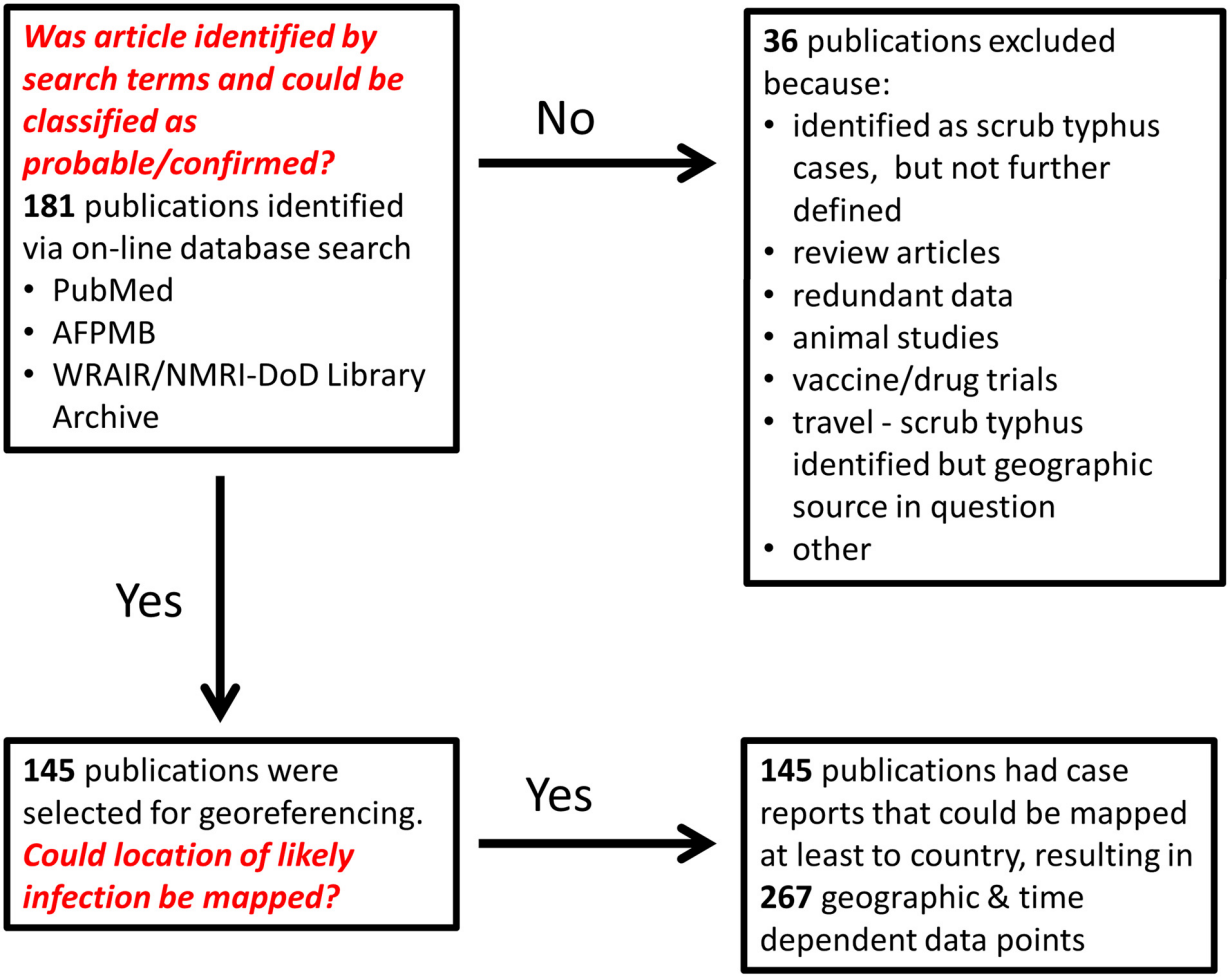
Flow chart used for scrub typhus case literature selection.

**Fig 3:**
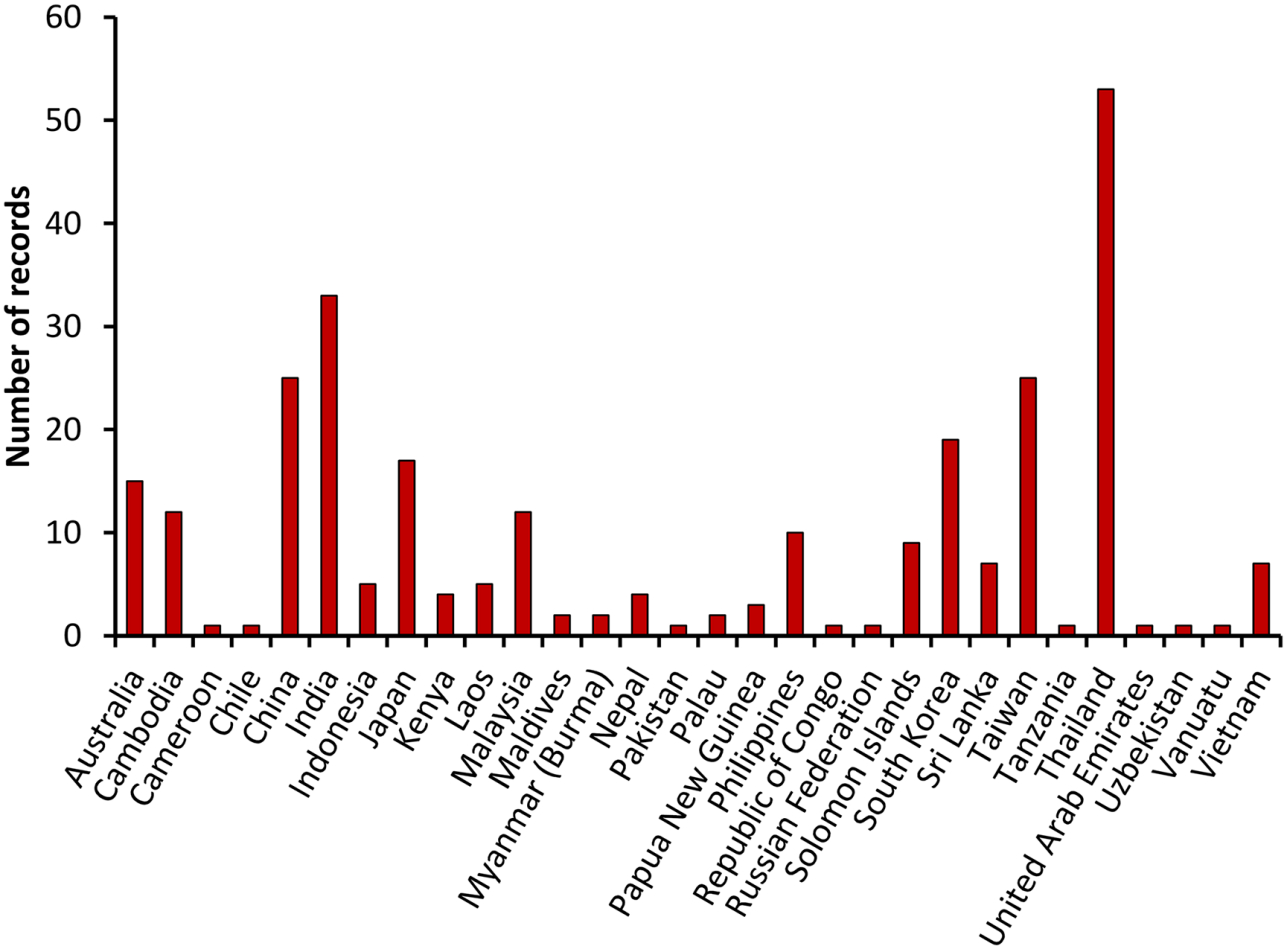
Frequency of report records of scrub typhus cases as reported by country.

**Fig 4:**
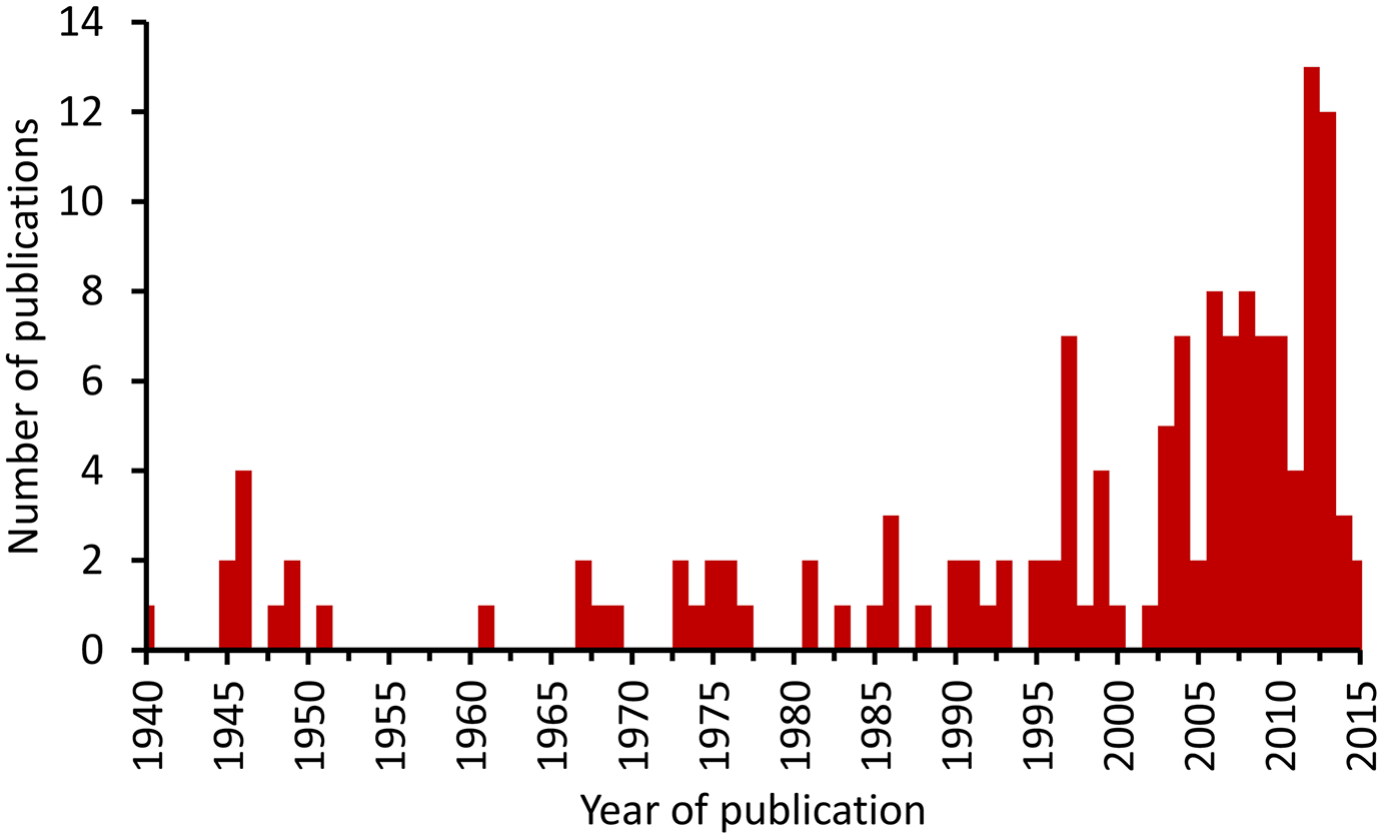
Publications reporting scrub typhus cases included in this study from published reports for the years 1940–2015.

As a result of the PubMed [10] and AFPMB [11] searches, 181 publications met the initial inclusion criterion, i.e., were identified using the literature search terms (Figs 1 and 2, S1 Data). Modified WHO scrub typhus case criteria that were used are described in Table 1 [12]. Separate records were included in the database for each distinct location in a reference. Publications which contained probable and confirmed cases from the same location were scored in separate records. A total of 267 data records were assigned from 145 scored publications for scrub typhus in 28 countries (Fig 1). The greatest portion of scored publications presenting confirmed and probable scrub typhus cases were from India (*n* = 24), Thailand (*n* = 22), People’s Republic of China (*n* = 14), Republic of Korea (*n* = 13), Taiwan (Republic of China) (*n* = 11), and Japan (*n* = 10) (Fig 3, Table 2). Few references from Russia, Mongolia, and Central China were noted. Of the 145 publications, 87 (60%) were published since 2000 (Fig 4). In this review, we noted that scrub typhus diagnosis was often based on single serum serological results that can often result in false positive reactions [16,17]. Thus, we chose to adapt the WHO Recommended Surveillance Standards by requiring for serologically confirmed cases at least a 4-fold rise in titer (Table 1) [12].

**Table 2:** Published cases of scrub typhus in humans from studies conducted since 1940 by country.

Country	Report records	Publications	Confirmed Cases^a^	Probable Cases
Australia	15	5	58 (59)	40
Cambodia	12	2	124 (100)	0
Cameroon^b^	1	1	1 (100)	0
Chile	1	1	1 (100)	0
China	25	14	7,836 (97)	242
China (Taiwan)	25	11	95 (48)	104
Congo, Republic of ^b^	1	1	1 (100)	0
India	33	24	357 (19)	1,510
Indonesia	4^c^	4	3 (27)	8
Japan	17	10	63 (5)	1,065
Kenya^b^	4	1	0 (0)	57
Korea, Republic of	19	13	553 (74)	191
Laos	5	4	80 (61)	52
Malaysia	11^c^	6	147 (60)	100
Maldives	2	1	2 (14)	12
Myanmar (Burma)	2	2	0 (0)	105
Nepal	3^c^	2	5 (10)	46
Papua New Guinea	3	3	1 (4)	25
Pakistan	1	1	3 (100)	0
Palau	2	1	6 (100)	0
Philippines	10	3	28 (56)	22
Russia	1	1	1 (100)	0
Sri Lanka	7	6	35 (44)	45
Tanzania	1^b^	1	1 (100)	0
Thailand	53	22	313 (53)	273
United Arab Emirates	1	1	1 (100)	0
Uzbekistan	1	1	0 (0)	7
Vietnam	7	6	120 (100)	0
Total	267	148^d^	9,835 (72)	3,904

^a^Brackets show percentage of scrub typhus cases scored as “confirmed.”

^b^Case confirmed or probable but country of acquisition may not be.

^c^Excludes seroepidemiology publications listed in Table 3.

^d^Three publications included two or more countries.

### Online access to VectorMap

**Fig 5:**
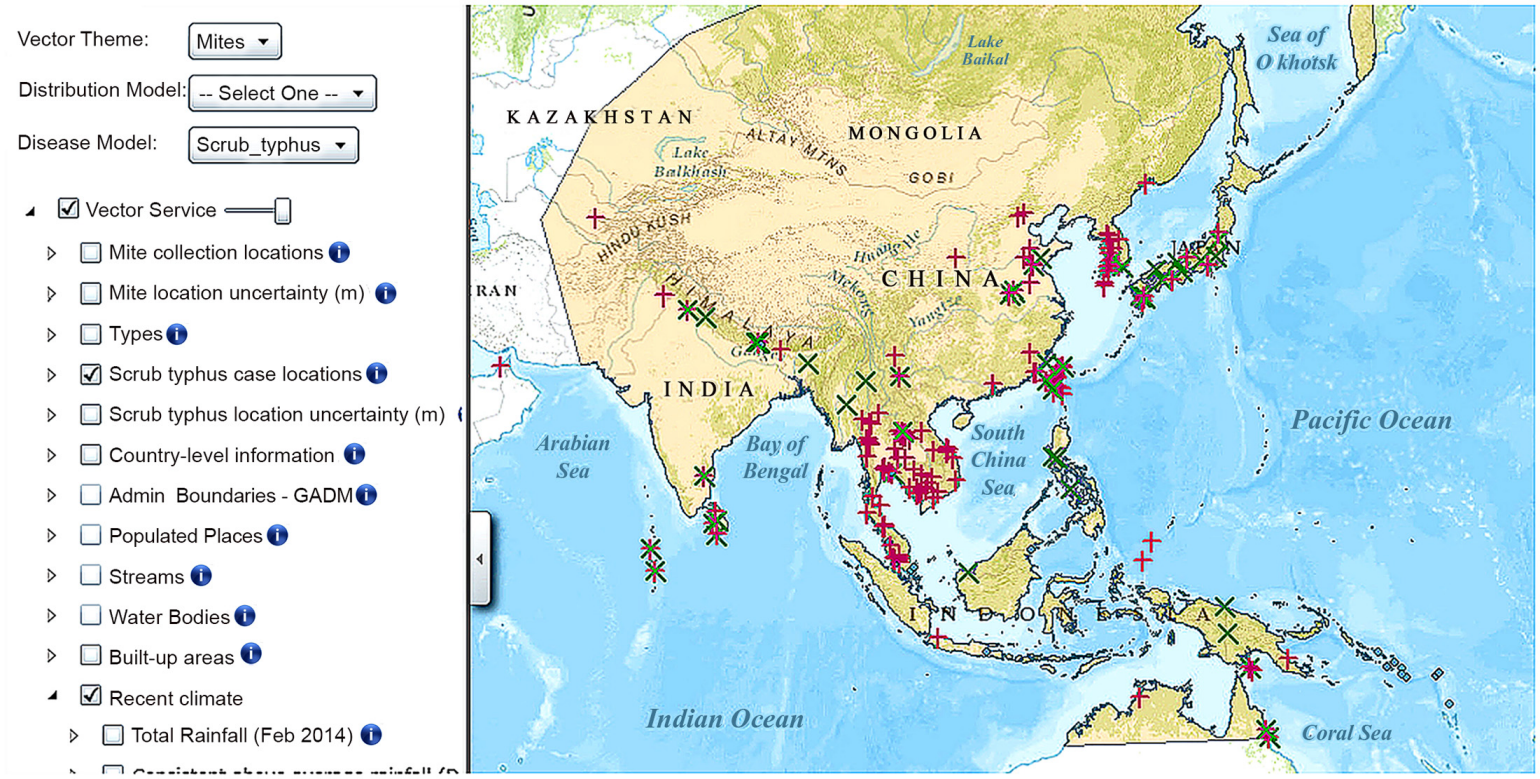
Opening screen of the scrub typhus VectorMap web application showing scrub typhus cases. Map area in orange shows traditional endemic area [18]; red “+” indicates site of confirmed cases, green “x” indicates site of probable cases, blue dots indicate serosurveys of scrub typhus positive sites.

The default display in the VectorMap viewer is “Vector Theme: Mosquitoes,” so the user has to select “Vector Theme: Mites” from the drop down list. To focus on case reports, deselect “Mite collection locations” and select “Scrub typhus case locations.” This will display the location of reports for probable and confirmed scrub typhus cases as well as serosurveys. Clicking on the small triangle next to the layer reveals the symbology (go to www.vectormap.org to download the VectorMap tutorial). Clicking on any defined scrub typhus case location will result in the display of all (30) fields of information for that record, including the associated “source” reference. A layer, “Scrub typhus location uncertainty (m),” is available to see the extent in meters of a circle that defines the area of the likely site of transmission. Fig 5 shows what the mite map service on VectorMap looks like. Under “Disease model,” a layer is available showing the endemic extent of scrub typhus (redrawn from [18]). Alternatively, an online map that does not require Silverlight can be seen at http://www.vectormap.org/Project_ScrubTyphus.htm. Average coordinate uncertainty was 100,797 m (*n* = 280) and ranged from 500–1,200,000 m. From the 280 records, 189 were classified as confirmed, 78 were probable (*n* = 267), and 13 were from serosurveys (Table 3). When both “probable” and “confirmed” cases are observed in a single study, separate records were generated, and the red “+” and green “x” are viewed superimposed in VectorMap (Fig 5).

**Table 3:** Published human seroepidemiological reports since 1981 of *Orientia tsutsugamushi*-specific antibody.

Country	Report record	Publications	Total sera tested^a^	Positive sera
Indonesia	1	1	464 (1.2)	6
Malaysia (Sabah)	1	1	837 (0.8)	7
Nepal	1	1	188 (9.2)	19
Solomon Islands	9	1	335 (32.5)	161
Vanuatu	1	1	72 (15.3)	13
Total	13	5	1,896 (9.8)	206

^a^Parentheses show percent positive

## Discussion

This examination of case data compiled from the literature and displayed on VectorMap demonstrates the dramatic geographical and topological range of scrub typhus case investigations throughout the last 70-plus years. This range includes the pre- (effective) antibiotic era of World War II, with thousands of cases in soldiers and hundreds of deaths reported [8], to the present day, when US military interest remains high [19–21]. Our examination underscores the difficulty in identifying the actual distribution and incidence of the human disease, for which there are several reasons. Publications usually do not give precise location information for the likely site of transmission, which resulted in large uncertainty estimates (up to 1,200 km in radius). Thus, vague regional descriptions (e.g., Northern New Guinea), or country and state or province information only are given. Using the likely patient catchment area of a hospital results in lower estimates of spatial uncertainty for civilian cases, but this becomes problematic for military hospitals that receive patients airlifted over a potentially larger area. The value in having an error estimate is that it allows the database user to determine how accurate the georeference is, affecting decisions such as what spatial resolution of remotely sensed data is appropriate for spatial modeling of scrub typhus occurrence data. Each paper also needed to be judged according to the likelihood of the clinician’s diagnosis. Even when modern diagnostic tools were available, we were dependent on the clinician’s suspicions, the differential diagnosis, and the endemicity of the disease [22]. The objective laboratory confirmation becomes paramount. In order to accurately score cases identified in the literature, we found that several different serologic and direct agent detection tests for laboratory confirmation of diagnosis were used (S1 Data, “Identification Method For Parasite”).

To accurately score cases, it was necessary for us to define both the clinical and laboratory criteria (Table 1), as scrub typhus is often confused with other diseases with similar presentations. The difficulty in diagnosing scrub typhus as a distinct disease is due to the inability to isolate and culture rickettsiae or to distinguish infection with *O*. *tsutsugamushi* from other rickettsiae; this has been recently discussed in a review of strategies for detecting rickettsiae and/or diagnosing rickettsial diseases [23]. We noted that a range of serological methods and endpoint criteria were used by investigators in the 145 publications reviewed. Introduction of the Weil-Felix test, first for typhus and spotted fevers [24] and subsequently for scrub typhus itself (Weil-Felix OX-K), [25] greatly facilitated diagnosis. Although relatively insensitive, the test has shown a specificity of up to 0.97 [11,26–28]. In our review, we found that the Weil-Felix test is still used at many locations to diagnose scrub typhus. For the purposes of this analysis, we chose to use it to score probable cases (single sera) or confirmed cases (4-fold rises in titer) when considered in conjunction with clinical presentation (Table 1). The next most common assay we encountered for serodiagnosis was the indirect fluorescent antibody assay (IFA) test. First developed in 1963 [29], its modification has become the “gold standard” [16,30], and the closely related immunoperoxidase (IIP) test reports similar accuracy [27]. WHO recommends these assays as the serological methods of choice for diagnosis of scrub typhus [31]. They concluded that confirmed diagnosis should be based upon seroconversion, i.e., ≥ 4-fold rise in IFA titer. In our examination of references, we also noted the extreme variation in IFA methods and cutoff values. Still, we believe the correlation of clinical presentation in addition to a positive serological value could be scored as a “probable” case, and in combination with a ≥ 4-fold rise in serological titer could reasonably be scored as a “confirmed” case.

In the more recent publications, newer methods such as polymerase chain reaction assay (PCR) [32], nested PCR assay [33–34], and quantitative real-time PCR (qPCR) assay [35] were used to confirm diagnosis. The PCR methodologies using blood samples show a very high specificity (99.7%) but generally low sensitivity (44.8%) [36]. A scrub typhus qPCR assay was found to have a sensitivity of 63.0% (17/27) when compared to serologically proven scrub typhus cases, a higher sensitivity than culture (seven of 17 isolate-negative specimens). Seropositive samples were positive by qPCR, and four of seven serum samples were positive by qPCR [37]. In contrast to the difficulties of obtaining PCR-positive blood or serum samples, biopsies of eschars have shown a much higher sensitivity: 86% in a Korean study [38] and 79.5% recently reported in a multi-site Indian study [39]. An eschar may not be detected by the patient or health care provider or may not exist because of immunity developed by prior infections [40]. When direct agent detection methodologies were used in the articles we evaluated, it was much easier to score cases as “confirmed,” but, by far, confirmation using paired sera serology was the most common laboratory method employed.

Unlike vector or rodent host studies that can be narrowed down to specific geographic areas, location information for cases was often limited to the clinic or hospital where the scrub typhus patient presented. This may be some distance away from where the infected chigger bite actually occurred. “Locality” was used to establish latitude and longitude (S1 Data). Often, references identified two or more localities with different sets of patients and were scored separately. The clinic or hospital where a febrile patient presented is not likely the site of exposure, but usually it is the only site noted in the reference. This is a limitation of the current study, and it points to an area where possible improvements in reporting could be made. Because of the usually rural nature of the disease, we assumed most patients presented at their local health clinic, typically within 10 km [40–41]. Data gathering for new cases and for additional chigger location data is underway (Dr. Paul Newton, email of May 11, 2015 email to DJK from PN; unreferenced; subject, chigger mapper; see Acknowledgments). We believe infected mite and host data will correlate spatially with patient presentation at local health care facilities [21,42]. Mite data in VectorMap represent published collections and testing for *O*. *tsutsugamushi* vectors (primarily *Leptotrombidium* spp.), hosts (primarily rodents and shrews), and testing for the rickettsiae. Pathogen data in VectorMap note the isolation of *O*. *tsutsugamushi*, identification method, dates of the collection, collection sites, tester organization, etc. While not yet as extensive as the tick, sand fly, and mosquito map services within VectorMap, mite and scrub typhus data are being added as studies are published. The recently added reservoir theme in VectorMap records rodents and their blood pathogens, showing the location of infected animals and mites that will correlate to the relative risk to people working in those areas. Clinicians authoring scrub typhus case reports are encouraged to gather data about where and when patients likely became infected, what they do for a living, and other epidemiologically important factors. This might point future investigators to the source of infections.

VectorMap has not been the only tool used to plot cases of scrub typhus or other febrile illnesses, including malaria. Acestor et al. [43] recently mapped suspected and confirmed non-malaria cases extracted from the literature and published between 1986 and 2011, focusing on the Mekong River region of Southeast Asia. The accuracy of the diagnoses is unclear, as the authors also depended upon the published data; however, “typhus,” i.e., scrub and murine, was second only to dengue as the most frequent pathogen reported in that review. We believe it to be important to more firmly establish the diagnosis, and thus we were somewhat conservative in scoring the diagnoses (Table 1).

The present study finds that scrub typhus mapping would benefit tremendously from more epidemiological detail from future case reports. We recommend that authors, editors, and reviewers be encouraged to request or require more information, especially the precise location description where the cases were likely acquired (ideally latitude and longitude from a GPS or other geolocation method) and the exact date of fever or presentation at the clinic. That information is often available to the attending physician, who can query patients on the spot. Inclusion of these data in scrub typhus case reports might encourage follow-up vector and reservoir studies such as mite flagging, “black plate” chigger collection, or host trapping, in order to more clearly define areas of high scrub typhus risk to humans [40].

Scrub typhus is but one of a host of febrile diseases endemic to the Asia-Pacific Rim (Fig 1). As more accurate diagnostic assays are being developed, interest in other rickettsioses is rising [8]. One example notes that patient serosurveys performed in 2006, 2007, and 2011 in two distinct regions of Thailand reported distinct reactivity to multiple rickettsioses and ehrlichioses [44].

The actual distribution of scrub typhus is poorly known globally, with few attempts at a global map. The map of endemic areas in Kelly et al. [18] is a recent attempt at a range map but lacks detail. Our map of case distribution is an attempt to further refine knowledge of spatial distribution and highlights some exceptions to the general pattern given in Kelly et al. [18], e.g., the case from Chile [9]. There is a need for a more precise map of scrub typhus disease risk, and data from infected chiggers and hosts may provide the collection data records needed; the uncertainty of these data should be low because of the limited home range of the rodent hosts. For example, the deer mouse *Peromyscus maniculatus* was shown to have a home range of 100 m, although many factors can influence movement, and a subset of itinerant rodents is possible [45]

Advances in our understanding of the ecology and distribution of scrub typhus are jeopardized by the decline in traditional chigger taxonomic expertise but improved by new molecular vector and pathogen detection methods. The results of the literature review show an apparent increase in the number of studies reporting scrub typhus, with the highest number (14) during 2013 (Fig 4). Reports dealing with scrub typhus are greatest in Thailand, followed by India, China and Taiwan, and South Korea and Japan. This may reflect endemicity or the experience and resources available to study and report on this disease. The compilation and dissemination online, via VectorMap, of locations where chiggers and scrub typhus cases have been recorded is an encouraging development for understanding how environmental, ecological, and climatic covariates relate to transmission and could assist with spatial modeling efforts to more accurately define the range of the disease. Improvements in the standard of diagnosis and in the georeferencing of likely transmission sites would assist efforts such as VectorMap to serve high resolution data and would allow modelers to use these data to predict areas of risk for contracting scrub typhus, e.g., using spatial modeling methods to more accurately define the range of the disease.

In summary, this survey presents the most comprehensive snapshot to date of the distribution of human scrub typhus. Vector Map itself is an online, user-accessible document for which new and historical publications will continue to be reviewed and case data added. It will be updated by users and refereed in a sustainable manner by the program monitor to provide an ever-more detailed picture of the distribution of the disease. The VectorMap webpage gives details and an email address where data and communications can be sent. Apparently, scrub typhus is either spreading or has been present all along but is an unrecognized cause of morbidity and mortality. We believe the historic and current information provided by Vector Map will be of value to health care workers, such as those involved with vaccine trials or conducting miticide ground spraying to reduce disease risk, and to clinicians who must diagnose and treat cases, sometimes in regions rarely reporting the disease. The value of mapping future outbreaks will be enhanced using advanced direct agent detection systems such as qPCR, especially as these capabilities become cheaper to manufacture and use. Future studies will hopefully receive increased funding from donor organizations and will involve ever-improving collaboration among national and international health agencies, greatly facilitated by the ever-growing ease of online disease monitoring.

**Key Learning Points**

- Scrub typhus is a potentially fatal disease of the Asia-Pacific Rim that is often misdiagnosed due to lack of accurate, user-friendly diagnostic tools.
- There is an increasing need to apply established standards to identify and track scrub typhus cases.
- VectorMap provides a tool for accurately pinpointing cases and developing risk assessments.
- As new cases are diagnosed using more accurate tools, data can be added to the interactive VectorMap database.
- Accurately identifying endemic areas and correctly diagnosing cases will result in improved health care.

**Top Five Papers**

1. Paris DH, Shelite TR, Day NP, Walker DH. Unresolved problems related to scrub typhus: a seriously neglected life-threatening disease. Am J Trop Med Hyg. 2013;89: 301–307. doi: 10.4269/ajtmh.13-0064.
2. Kelly DJ, Fuerst PA, Ching W-M, Richards AL. Scrub typhus: the geographic distribution of phenotypic and genotypic variants of *Orientia tsutsugamushi*. Clin Infect Dis. 2009;48: S203-230.
3. Kelly DJ, Richards AL, Temenak J, Strickman D, Dasch GA. The past and present threat of rickettsial diseases to military medicine and international public health. Clin Infect Dis. 2002;34: S145-169.
4. Foley DH, Wilkerson RC, Birney I, Harrison S, Christensen J, Rueda LM. MosquitoMap and the Mal-area calculator: new web tools to relate mosquito species distribution with vector borne disease. Int J Health Geogr. 2010;9:11–18.
5. Foley DH, Wilkerson RC, Rueda LM. Importance of the “what,” “when,” and “where” of mosquito collection events, J Med Entomol. 2009;46: 717–722.

## Supporting Information

S1 Data VectorMap spreadsheets of 4/7/15: identification of data fields. (XLSX)
